# Advancement of Phenoxypyridine as an Active Scaffold for Pesticides

**DOI:** 10.3390/molecules27206803

**Published:** 2022-10-11

**Authors:** Yanfei Liu, Bin Fu, Yanjun Xu, Bo Ren, Zhaohai Qin

**Affiliations:** 1 College of Science, China Agricultural University, Beijing 100193, China; 2 Department of Laboratory Animal Science, Peking University Health Science Center, Beijing 100191, China

**Keywords:** phenoxypyridine, pesticide, active, structure-activity relationships

## Abstract

Phenoxypyridine, the bioisostere of diaryl ethers, has been widely introduced into bioactive molecules as an active scaffold, which has different properties from diaryl ethers. In this paper, the bioactivities, structure-activity relationships, and mechanism of compounds containing phenoxypyridine were summarized, which may help to explore the lead compounds and discover novel pesticides with potential bioactivities.

## 1. Introduction

**Figure 1:**
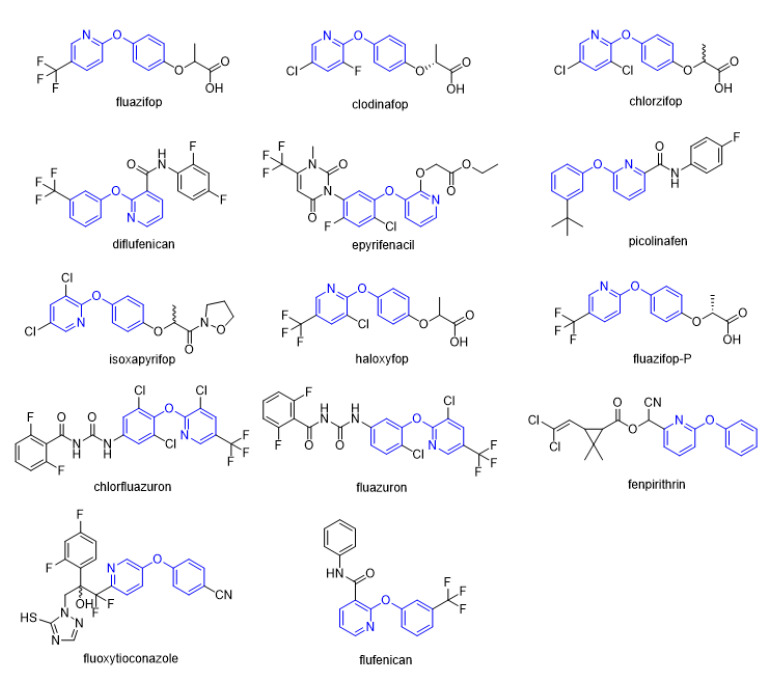
Selected commercial pesticides containing phenoxypyridine.

Diaryl ether [1] is an important active fragment in pesticide molecules, which has good lipid solubility, metabolic stability, cell membrane penetration, sufficient molecular flexibility [2], and can improve biological activity and photostability. So far, the structure of diaryl ether has been widely studied and applied, such as aryloxyphenoxypropionate herbicides, pyrethroid insecticides [3], and triazole fungicides. Pyridine [4], as a nitrogen-containing heterocyclic ring, plays an important role in agrochemicals, and its derivatives have a wide range of biological activities. The hydrophobicity (one of key properties affecting biological activity) of pyridine is significantly higher than that of the benzene ring [5]. Meanwhile, pyridine is an ionizable polar aromatic compound, which can optimize solubility and bioavailability of the lead compound [6]. Replacing the benzene ring with a pyridine ring [7] can usually increase the π-π stacking probability of the target molecule [8] and improve the biological activity (Table 1). Therefore, phenoxypyridine may have properties that are different from or even superior to those of diphenyl ether. The phenoxypyridine structure has been widely used in the molecular structure of pesticides. At present, there are many commercial pesticides containing the phenoxypyridine structure, as shown in Figure 1. The active skeleton is of great significance for the creation of new pesticides and there is no report on the summary of phenoxypyridine compounds. In this paper, we will summarize the research about the relevant phenoxypyridine derivatives in the pesticide field in the last ten years.

## 2. Herbicides Containing Phenoxypyridine Scaffold

### 2.1. Acetyl CoA Carboxylase Inhibitors

Acetyl CoA carboxylase (ACCase) inhibitors [16,17] target ACCase [18] to inhibit fatty acid synthesis in gramineae plants. There are two classes of ACCase inhibitors: aryloxyphenoxypropionate [17] (AOPP or fop) and cyclohexanediones (CHD or dim). Aryloxyphenoxypropionate herbicides [19] occupy an important position in the world herbicide market which have characteristics of high efficiency, low toxicity, crop safety, and so on. In 1976, Ishihara discovered that the compound that was obtained by substituting the benzene ring on one side with a pyridine ring had higher herbicidal activity and launched the first aryloxyphenoxypropionate herbicide containing phenoxypyridine–pyrifenop [20]. Since then, extensive research on herbicides containing phenoxypyridine had been initiated.

**Figure 2:**
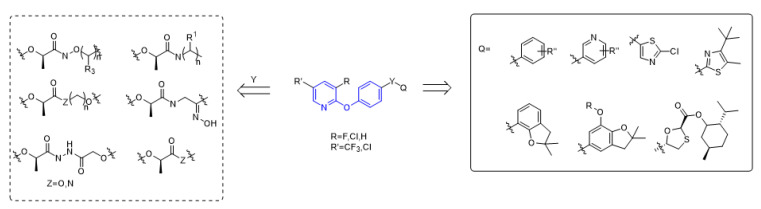
The general structural formula of ACCase inhibitors.

The structure of aryloxyphenoxypropionate herbicides containing phenoxypyridine is shown in the Figure 2, in which part A is phenoxypyridine with different substitutions, most of which were electron withdrawing groups, such as F, Cl, Br, NO_2_, CN, and CF_3_; part Y is the linking arm, where conformation R [19] was the active ingredient of herbicide; and part Q are various heterocycles, both aromatic and non-aromatic (pyridine, thiazole, benzofuran, etc.).

**Figure 3:**
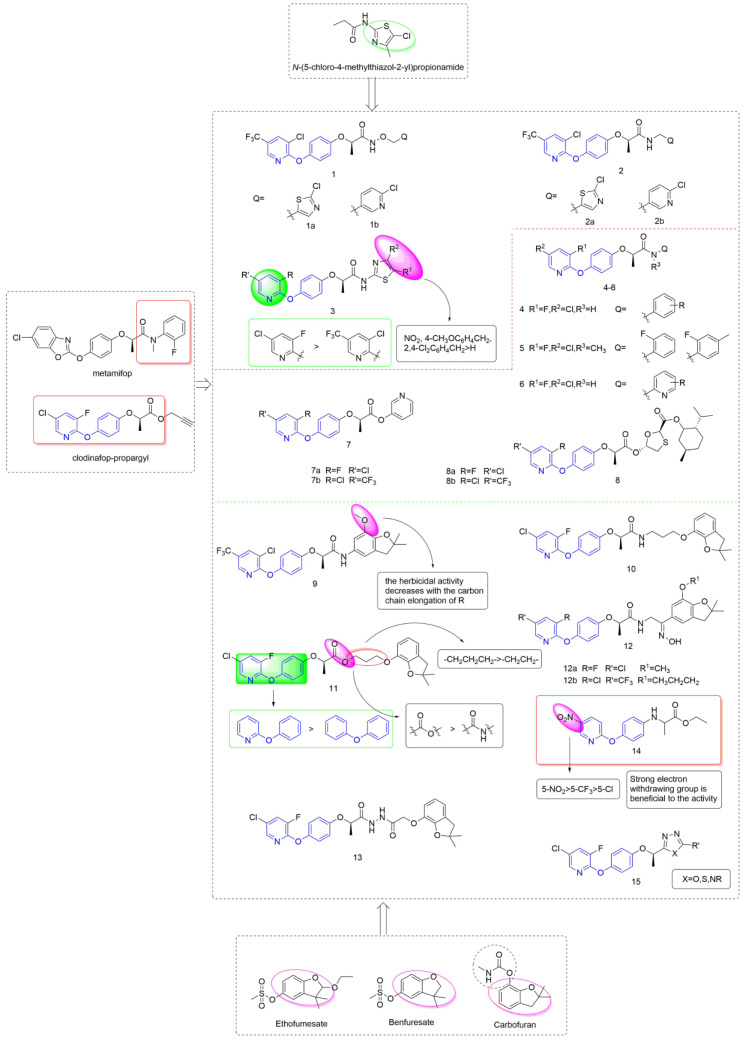
ACCase inhibitors containing phenoxypyridine.

Taking metamifop and clodinafop as the leader, phenoxypyridine was linked to various aromatic rings through different linking arms to obtain active molecules with different structures, as shown in Figure 3. The synthesis of newer aryloxy phenoxycarboxylic acid amide derivative Compound **1** that was equipped with arylalkoxy was reported by Huang et al. [21] and assessed for herbicidal activities. Compounds **1a** and **1b** all showed 100% control efficiency against *Digitaria sanguinalis* in post-emergence applications, even at doses as low as 37.5 g a.i/ha and 18.75 g a.i/ha. Likewise, Wang et al. [22] designed and synthesized compound **2** by introducing an arylalkyl group into the structure of aryloxy phenoxycarboxylic acid amide. The inhibitory activity of Compounds **2a** and **2b** against *Digitaria sanguinalis*, *Echinochloa crus-galli*, and *Setaria viridis* under post-emergence was 100% at a dose of 60 g ai/ha. At the same time, Compound **2b** was very safe for rice, and **2a** was slightly less than **2b**. The thiazole groups with different substituents were directly connected to an amido bond to yield Compound **3** [23], most of which showed a 100% inhibition rate against *Digitaria sanguinalis*, *Echinochloa crus-galli* and was comparable to metamifop. The effect of these compounds under post-emergence was slightly better than that under soil treatment, which could be used as post-seedling herbicide. The structure-activity relationship showed that 3-fluoro-5-clopyridine > 3-chloro-5-trifluoromethylpyridine; the order of influence of R^1^ groups was: NO_2_, 4-CH_3_OC_6_H_4_CH_2_, 2, 4-Cl_2_C_6_H_4_CH_2_>H.

Using clodinafop-propargyl and metamifop as a lead structure, Compound **4** was synthesized by Yang et al. [24] through active group splicing and exhibited high selective herbicidal activity against monocotyledonous grass weeds (*Beckmannia syzigachne (Steud.) Fern.*, *Polypogon fugax Nees ex Steud.,* and *Poa annua* L.) at 150 g/ha. The chlorine-substituted target compound showed higher inhibitory activity against *Polypogon fugax Nees ex Steud.* than the compound with fluorine substituted. The control effect of Compound **5** that was synthesized by Xiao et al. [25] against *Digitaria sanguinalis*, *Echinochloa crus-galli*, and *Setaria viridis* was 100% at the dose of 85 g ai/ha. Compound **6** [26] showed 100% inhibitory activity against *Echinochloa crus-galli* and *Setaria viridis* at the concentration of 15 g ai/ha, and was safe for rice. Liu et al. proposed that aryloxyphenoxyalkanoic acid ester analogues **7** [27] and **8** [28] showed selective herbicidal activity against monocotyledonous weeds (*Digitaria sanguinalis*, *Echinochloa crus-galli,* and *Setaria viridis*) with more than 90% efficacy in both post-emergence and soil treatment at 5 g/mu.

Lin et al. [29,30] integrated a benzofuran unit into the scaffold of aryloxy phenoxycarboxylic acid amide to yield Compound **9**. Compound **9** exhibited 100% control efficiency at the concentration of 2250 g/hm^2^ at both pre- and post-emergence applications. According to the SARs, substituents on pyridine had little effect on the herbicidal activity. The group of R plays a crucial role in herbicidal activities, and the herbicidal activity decreases with the increase of the carbon chain length of R. The aryloxyphenoxy propionamide could be linked with benzofuran via an alkoxy chain to give Compound **10** [31]. Compound **10** displayed a 98.7% inhibition rate against *Echinochloa crus-galli* whether with treatment by post-emergence or soil treatment at 25 g/mu. The linker between phenoxypyridine and benzofuran in Compound **10** was changed to an amido bond by Yan et al. [32], and the resulting compounds presented significantly better herbicidal activity against monocotyledonous weeds than dicotyledonous weeds. For monocotyledonous weeds, the herbicidal effect of post-emergence treatment was equivalent to that of pre-emergence treatment. Further analysis revealed that Compound **11** (100% inhibitory activity) exhibited better herbicidal activity than clodinafop-propargyl (89.9% and 84.7% inhibitory activity) against *Echinochloa crus-galli* either pre- or post-emergence application at 375 g.ai/ha.

The structure-activity relationship showed that the herbicidal activity of propionate derivatives somewhat exceeded that of propionamide derivatives. The results showed that increased lipid solubility was beneficial to the herbicidal activity of these compounds to a certain extent; the activity was significantly increased after the introduction of pyridine; in addition, the substitution in the pyridine ring had an important effect on the activity. For propionate derivatives, the activities of the compounds with n = 3 of alkyl chain were better than n = 2. However, for propionamide derivatives, increasing the length of the alkyl chain was less effective. In addition, it was confirmed by an enzyme activity test that **11** was a pro-herbicide [33], which acts in plants by hydrolyzing the ester into acid.

The structure of oxime was introduced to the skeleton by Hu et al. [34] to give Compound **12**. Further analysis revealed that Compound **12a** exhibited the highest herbicidal activity (100% inhibition rate) against *Digitaria sanguinalis* and *Echinochloa crus-galli* under soil treatment at a dose of 100 g/mu, and the control effect of Compound **12b** under post-emergence was 100%. The phenoxypyridine could be linked with benzofuran via acylhydrazine to give aryloxyphenoxypropionic hydrazide derivatives and the herbicidal activity was tested by Yang et al. [35]. At the dose of 75 g/hm^2^, Compound **13** showed greater than 90% inhibition against *Beckmannia syzigachne (Steud.) Fern.* Under soil treatment, close to 100% inhibition against *Eleusine indica* (L.) *Gaertn.* when used post-emergence, and a certain inhibition effect on dicotyledonous weeds.

Xu et al. [36] took haloxyfop-methyl as the lead compound, introduced the structure of aryloxyanilino group based on bioisosterism, and designed a series of compounds containing pyridoxyanilino propionic acid/ethyl acetate. The new compounds showed a certain herbicidal activity against *Echinochloa crus-galli*, and the IC_50_ of **14** was 27.692 mg/L, which was at the similar level to that of the control haloxyfop-methyl (26.959 mg/L). Preliminary structure-activity relationships revealed that the new compounds exhibited enhanced herbicidal activity with the introduction of the strong electron-withdrawing substituent nitro on the pyridine ring. Moreover, the bioactivity of the compound with an electron-withdrawing substituent at position 3 of the pyridine ring was higher than at position 5. This provides a novel structural skeleton for the study of this class of compounds. Compound **15**, which was reported by Kalhor et al. [37], showed fair to good activity, in which the 1,2,4 triazole structure contributed to the improvement of herbicidal activity and crop selectivity.

### 2.2. Protoporphyrinogen IX Oxidase Inhibitors

**Figure 4:**
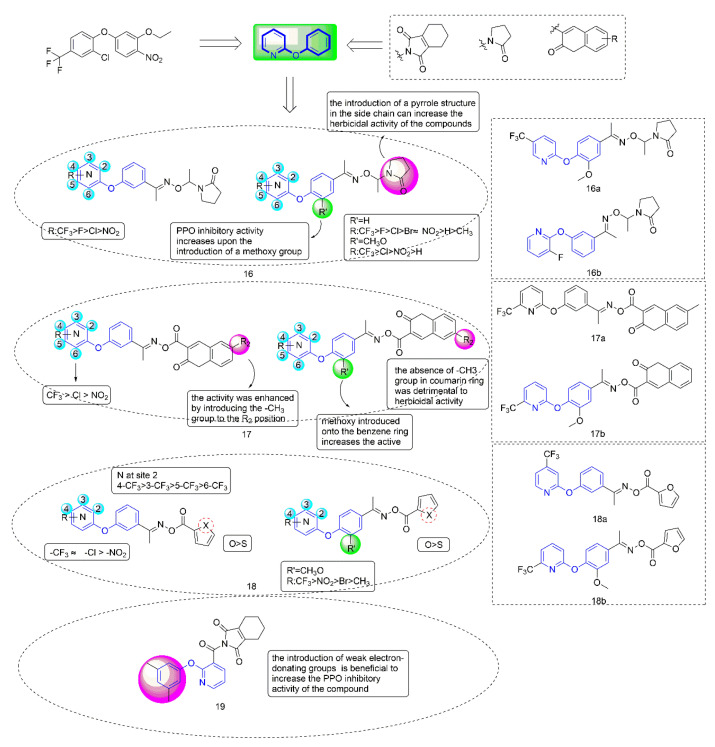
Protoporphyrinogen IX oxidase inhibitors containing phenoxypyridine.

Protoporphyrinogen oxidase (PPO) [38] is a key enzyme in the biosynthesis of chlorophyll and heme in plants and is one of the important targets for the creation of novel herbicides. At present, PPO-inhibiting herbicides mainly include diphenyl ethers, phenylpyrazoles, triazolinones, *N*-phenyl phthalimides, and diazoles [39]. Among these herbicides, diphenyl ethers (DPEs) [40] had been widely studied by researchers in the creation of novel pesticides due to their high efficiency, low toxicity, high selectivity, and simple synthesis process; Ye Fei’s team committed to the research and development of PPO inhibitors for a long time. Several series of compounds (Figure 4) containing phenoxypyridine had been designed, studied for greenhouse herbicidal activity, PPO inhibitory activity, crop selectivity, and structure-activity relationships (SARs). These studies fully confirmed that phenoxypyridine provided good herbicidal activity.

PPO inhibitors were known to compete with protogen IX by mimicking part of its structure, so they introduced pyrrolidone into the second ring side chain of diphenyl ether structure to simulate the three rings of protoporphyrinogen IX. Diphenyl ether derivatives with oxime substituents could significantly improve the herbicide activity and crop selectivity. Therefore, the Compound **16** series were designed and synthesized with the introduction of both oxime and pyrrolidone. Compounds **17** and **18** were synthesized by introducing coumarin and five-membered heterocycle, respectively.

When diphenyl ether was replaced by phenoxypyridine, the herbicidal activity and PPO inhibitory activity of the compounds were significantly increased, and herbicidal spectrum was significantly expanded. Most of the compounds showed strong PPO inhibitory activity in vitro, which was consistent with their herbicidal activity. Most compounds distinctly presented better inhibitory effects on dicotyledonous weeds than monocotyledonous weeds. Among them, the IC_50_ of Compound **16a** [41] (IC_50_ = 0.041 mg/L), **16b** [42] (IC_50_ = 0.0262 mg/L), **17a** [43] (IC_50_ = 0.01937 mg/L), **17b** [44] (IC_50_ = 0.01252 mg/L), **18a** [45] (IC_50_ = 0.032 mg/L), and **18b** [8] (IC_50_ = 0.0468 μmol/L) against PPO was consistent with or better than that of oxyfluorfen. At 150 g a.i./ha, Compound **16a** achieved 100% inhibition against *A. theophrasti* for post-emergence treatment. The herbicidal activity of Compounds **17a** and **17b** reached level A at 300 g a.i./ha^−1^.

The structure-activity relationship indicated that the herbicidal activity of the compounds with electron-withdrawing substituents on the pyridine ring was significantly higher than that of the compounds with electron-donating substituents. The introduction of the pyrrole structure in the side chain could significantly improve the herbicidal activity of the compound. The introduction of a coumarin ring at the para position of phenoxypyridine was shown to enhance the inhibitory activity of target compounds. Meanwhile, the type of substituents that were introduced on the coumarin ring had a significant effect on the herbicidal activity of the compound. Compounds containing furan rings showed better herbicidal activity than compounds containing thiophene rings. The most critical finding for Compound **18** was that the introduction of a trifluoromethyl group on the pyridine ring increased the inhibitory activity against PPO and varied when changing the substitution position.

The typical characteristic of PPO inhibitors, which were previously known as albino herbicides, is that the weeds are bleached and curled to death by inhibiting chlorophyll synthesis. Most weeds exhibited unique bleaching that was consistent with the symptoms following PPO herbicides application. Compared with the corresponding diphenyl ether compounds, the compounds containing phenoxypyridine could significantly reduce Ca and Cb contents of *A. retroflexus*, indicating that the compounds containing phenoxypyridine had a better bleaching effect. Multiple crops showed strong tolerance to **16a** (rice, peanut, and cotton), **16b** (rice, peanut, and cotton), **17a** (maize, cotton, and soybean), **18a** (rice, wheat, maize, and soybean), and **18b** (rice, wheat, maize, and soybean) at 300 g ai/ha. Field tests showed that the compound had a good inhibitory effect on weed growth. The amino acid residues PHE-392 and ARG-98 were important groups that were involved in the catalysis of porphyrins in organisms. Molecular docking results showed that compounds **16a**, **16b**, **17a**, **18a,** and **18b** acted more tightly on the active site than oxyfluorfen. Most of them form two hydrogen bonds with surrounding amino acid residue AGR-98.

Considering that *N*-phenyl-phthalimide herbicides [46] had the advantages of fast degradation rate, short residual time, and no pollution to the environment [47], Zhao et al. [48] introduced tetrahydrophthalimide to improve the selectivity and degradability of herbicides. Compound **19** had an IC_50_ value of 0.00667 mg/L against PPO, and exhibited similar herbicidal activity to oxyfluorfen. The structure-activity relationships indicated that the introduction of weak electron-donating groups on the benzene ring of the compounds was beneficial to increase the PPO inhibitory activity of the compounds. When the phenoxypyridine structure was replaced with phenylthiopyridine, the PPO inhibitory activity of the compound was significantly reduced. Similarly, most of the tested weeds bleached and died. In general, Compound **19** had a bleaching effect on weeds, acted more tightly on the active site, and showed higher safety and selectivity, making **19** a potential new herbicide candidate in the field.

### 2.3. Other Herbicides

**Figure 5:**
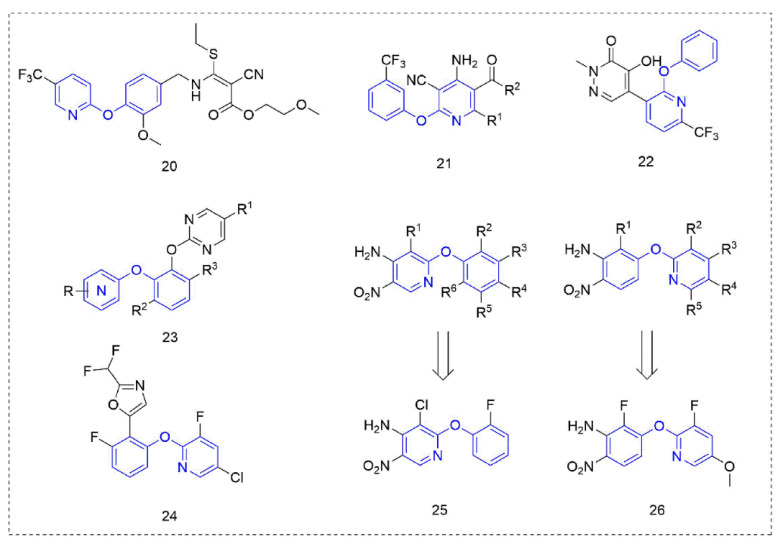
Other compounds with herbicidal activity containing phenoxypyridine.

Cyanoacrylate derivatives [49] are photosystem II (PS II) inhibitors [50], which can control weeds by interfering with electron transfer in the photosynthetic system of the plant, preventing photosynthesis. This special mechanism makes cyanoacrylate extremely safe for animals, in line with the requirements of the current social market for new herbicides. The compounds (Figure 5) that were obtained by linking the trifluoromethyl-substituted phenoxypyridine unit with cyanoacrylate skeleton showed good herbicidal activity. The herbicidal activity of the target Compound **20** [51] against *Digitaria sanguinalis*, *Echinochloa crus-galli*, *Abutilon theophrasti Medicus*, *Amaranthus retroflexus* L., and *Eclipta prostrata* (L.) L. was 100%.

The mechanism of phytoene desaturase (PDS) inhibitors [52] is to inhibit the catalytic action of phytoene desaturase in the biosynthesis pathway of carotenoids, and then inhibit plant photosynthesis and cause the plant to stop growing until it dies. Therefore, PDS inhibitors belong to carotenoid biosynthesis inhibitors, and the most obvious manifestation of plants that are treated are albino symptoms [53]. Compound **21** was designed by Zhai et al. [54] based on picolinafen and diflufenican and showed moderate herbicide activity against *Brassica campestris L* at a concentration of 100 mg/L. Compounds, where R^1^ was an electron-donating substituent, showed better activity than those with an electron-withdrawing substituent, and when R^2^ was a methoxy group, the activity was better than that of an ethoxy group.

The pyridazinone Compound **22,** reported by Syngenta [55], showed 80–100% activity against *Solanum nigrum* L. and *Amaranthus retroflexus* L. at 25 g a.i./ha. The bis(aryl)catechol derivatives **23** designed and synthesized by DuPont [56] had excellent inhibitory activity against a variety of weeds. The novel herbicidal phenoxypyridine compounds that were reported by Syngenta [57] showed improved properties compared to the known pyrimidine compounds—especially improving crop (soybean) selectivity. Compound **24** had significant effects on various weeds (*Lolium perenne*, *Solanum nigrum*, *Amaranthus retoflexus*, *Setaria faberi*, *Echinochloa crus-galli,* and *Ipomoea hederacea*) at a concentration of 500 g/ha. In 2020, two kinds of phenoxypyridine-containing compounds with herbicidal activity were discovered and reported by Bayer [58,59]. At 1280 g/ha, Compounds **25** and **26** showed more than 90% activity against a variety of weeds whether with treatment by preemergence (*Amaranthus retroflexus*, *Stellaria media,* and *Veronica persica*) or post-emergence (*Poa annua*, *Amaranthus retroflexus*, *Stellaria media,* and *Bassia scoparia*).

## 3. Fungicides and Bactericides Containing Phenoxypyridine Scaffold

### 3.1. Complex I Inhibitors

**Figure 6:**
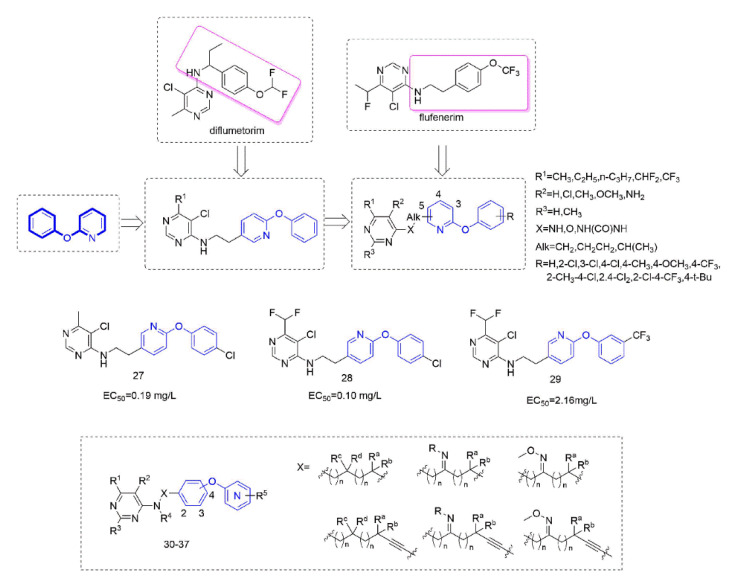
Complex I inhibitors containing phenoxypyridine.

Diflumetorim is a member of aminoalkylpyrimidines [60] targeting mitochondrial complex I (MET I) [61] which has a unique mode of action that is different from the MET I inhibitor acting as insecticide [62]. Therefore, it has no cross-resistance with existing traditional fungicides and is safe for non-target organisms. Liu and co-workers devoted to the research of pyrimidine amine compounds (Figure 6), and the fungicidal activity of the compounds that were synthesized by introducing a phenoxypyridine structure was significantly improved.

Several series of aminoalkylpyrimidine analogs containing phenoxypyridine fragments were designed and synthesized to study the control effect of cucumber downy mildew and the structure-activity relationships. The structure-activity relationships indicated that the compounds with Alk = CH_2_CH_2_ exhibited higher fungicidal activity than the corresponding analogues with Alk = CH_2_. When the pyrimidine group was attached to the pyridine ring at position 3 or 4, the fungicidal activity of these compounds decreases sharply. The substitutions of R^1^ and R^2^ on the pyrimidine ring were critical to exert fungicidal activity, while R^3^ does not contribute significantly to enhance fungicidal activity. Compounds containing phenoxypyridine had better activity than those containing diphenyl ether. Among them, the activity of Compounds **27** [63] (EC_50_ = 0.19 mg/L) and **28** [15] (EC_50_ = 0.10 mg/L) against cucumber downy mildew was significantly higher than that of diflumetorim (EC_50_ = 23.06 mg/L). In addition, the researchers found that the introduction of phenoxypyridine led to a significant increase in the activity against southern corn rust (SCR). The newly designed Compound **29** [64] displayed an EC_50_ value of 2.16 mg/L, which was superior to the commercial control diflumetorim. (EC_50_ = 53.26 mg/L). In the past few years, BASF had reported several aminoalkylpyrimidine derivates **30–37** [65,66,67,68,69,70,71,72]. As shown in the Figure 6, phenoxypyridine was linked to aminoalkylpyrimidine in various link arms resulting in some molecules with good protective fungicidal activity.

### 3.2. Complex III Inhibitors

**Figure 7:**
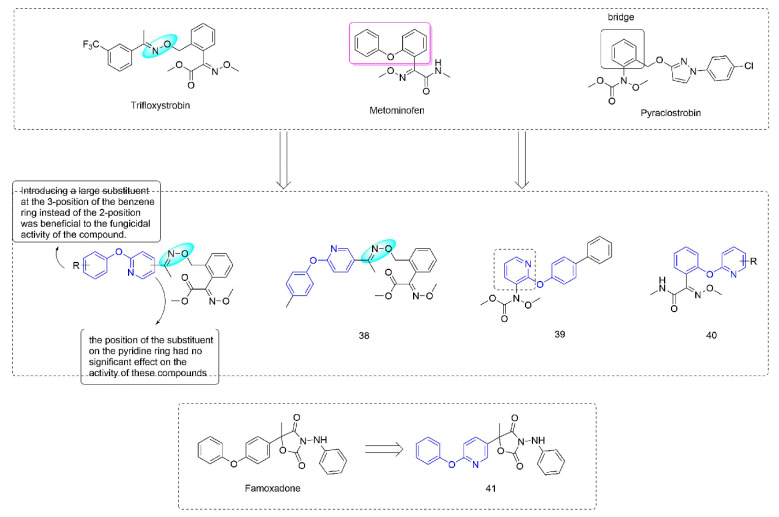
Complex III inhibitors containing phenoxypyridine.

Strobilurin [73] were derived from strobilurin A [74], a natural antibiotic with bactericidal activity, and were a kind of agricultural fungicide with great development potential and market vitality [75,76]. Strobilurins act on the Qo site of mitochondrial electron transport chain complex III and are also known as Qo site inhibitors. Some strobilurin derivatives containing phenoxypyridine are shown in Figure 7. A series of strobilurin analogues containing oxime ether structures were synthesized through introducing a phenoxypyridine group by Liu et al. [77]. Most of the compounds showed good fungicidal activity, with a significantly broadened antifungal spectrum compared to the compounds containing diaryl ether previously that were reported by BASF [78], among which the EC_50_ of **38** [79] against *Sclerotinia sclerotiorum* could reach 0.47 μg/mL. The trans-configuration was a dominant configuration. The disubstituted compounds on the benzene ring were less active than the monosubstituted compounds. Wang et al. [80] constructed a phenoxypyridine structure by modifying the bridge structure in strobilurins. The newly synthesized compounds showed certain fungicidal activity, among which the IC_50_ values of **39** against *Botrytis cinerea* and *Sclerotinia sclerotiorum* could reach 0.98 μg/mL and 0.64 μg/mL, respectively. The alkoxyiminoacetamide derivatives **40**, reported by Hayase et al. [81], had good activity against a variety of pathogenic fungi (*Botrytis cinerea*, *Pseudoperonospora cubensis*, *Sphaerotheca fuliginea*, and *Pyricularia oryzae*). The pyramoxadone **41**, developed by Qin et al. [9], had strong inhibitory activity against a variety of plant pathogens (*Rhizoctonia solani*, *Pythium aphanidermatum*, *Pyricularia grisea*, *Phytophthora capsica,* and *Phomopsis asparagi (Sacc.) Bubak*). Meanwhile, the IC_50_ value of the inhibitory activity of pyramoxadone against sporangium release of *Phytophthora capsica* was 13.85 μg/mL.

### 3.3. Sterol Biosynthesis Inhibitors

**Figure 8:**
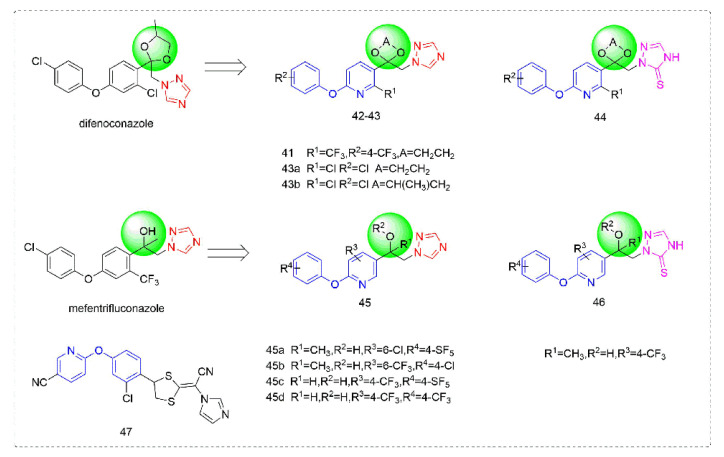
Sterol biosynthesis inhibitors containing phenoxypyridine.

Triazole fungicides are a new type of fungicide with broad spectrum, high efficiency, low residue, long effect, good systemic translocation, and both protective and curative effects. Triazole fungicides belong to ergosterol biosynthesis inhibitors, which mainly inhibit the activity of sterol 14α-demethylase in sterol biosynthesis to achieve fungicidal effects [82,83]. The triazole derivatives (Figure 8) that were synthesized by Bayer exhibited good protective activity against a variety of pathogenic fungi (*Puccinia recondite*, *Sphaerotheca fuliginea*, *Uromyces appendiculatus*, and *Blumeria*). The protective activity of compound **42** [84] against *Septoria tritici* reached 100% at 100 mg/L. The ED_50_ of Compound **43a** [11] against *Alternaria* and *Pyricularia oryzae Cav.* reached 0.12 mg/L and 0.56 mg/L, respectively, while **43b** [11] was 2.7 mg/L, 0.008 mg/L and 1.2 mg/L against *Botrytis cinerea*, *Sphaerotheca fuliginea* and *Pyricularia oryzae Cav*. **44a**, **45b**, **42c**, and **45d** [14] showed 100% protective activity against *Sphaerotheca* at 10 mg/L. Compound **46** [85] had 90–100% control effect against various pathogens at 500 mg/L. Some imidazole derivatives (such as clotrimazole, ketoconazole, imidazole, and oxazole) also inhibited 14 α-demethylase (CYP51). Jeanmart et al. [86] reported a series of novel compounds that were based on the modification of imidazole-based ketene dithioacetals lanoconazole and luliconazole. Compound **47** with the ketene dithioacetal [87,88] scaffold showed certain fungicidal activity, with 79% inhibitory activity against *Alternaria solani.*

### 3.4. Succinate Dehydrogenase Inhibitors

**Figure 9:**
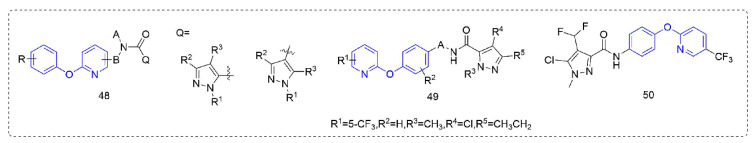
Succinate dehydrogenase inhibitors containing phenoxypyridine.

Succinate dehydrogenase inhibitors are a class of fungicides with a long history of development, accounting for a considerable proportion of fungicides. Succinate dehydrogenase inhibitors mainly bind to the ubiquinone pocket of SDH and mainly affect the electron transfer of the respiratory chain, to inhibit the growth of pathogenic fungi and eventually lead to death. Most of the pyrazole amide, such as Compounds **48** (Figure 9), that were designed and synthesized by Guan et al. [89] showed good protective activity against *Pseudoperonospora cubensis (Berk.et Curt.) Rostov.*, *Blumeria graminis*, and *Puccinia sorghi* in addition to certain insecticidal activity. The control effect of Compound **49** [90] against *Pseudoperonospora cubensis (Berk.et Curt.) Rostov.* was 100% at 12.5 ppm, and the control effect in the field was also better than that of dimethomorph. The activity of Compound **50** which was synthesized by Sun et al. [91] against *Pyricularia grisea* was significantly better than that of diphenyl ether and other skeleton compounds, with an EC_50_ value of 2.286 μg/mL, similar to fluxapyroxad (2.101 μg/mL), more than eight-fold higher than isopyrazam and more than 15-fold higher than the aminopyralid boscalid. Preliminary mechanistic studies suggested that these compounds may not be SDH inhibitors, but inhibited fungal growth by inducing plant defense responses.

### 3.5. Other Fungicides and Bactericides

**Figure 10:**
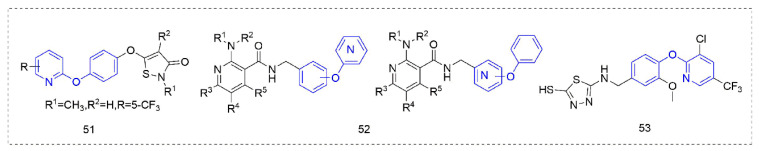
Other compounds with fungicidal or bactericidal activity containing phenoxypyridine.

Some other types of compounds containing phenoxypyridine structures with fungicidal or bactericidal activity are summarized in Figure 10. Phenoxypyridine was linked to isothiazolinone, resulting in Compound **51** [92] with good control effects on *Blumeria graminis*, *Botrytis cinereal,* and *Pyricularia grisea* at low doses. The introduction of chlorine at 4-position of isothiazolinone made the compound lose its inhibitory effect on *Botrytis cinereal* [93]. The Compounds **52** that were synthesized by Nippon Soda Co., Ltd. (Tokyo, Japan) [94] showed more than a 75% control effect against cucumber gray mold at 500 mg/L and did not cause any damage to the plant.

A series of vanillin derivatives [95] containing 1,3,4-thiadiazole moiety were synthesized and their antibacterial activities were evaluated against *Xanthomonas oryzae pv. oryzae* (*Xoo*) and *Xanthomonas oryzae pv. oryzicola* (*Xoc*). Among them, Compound **53** [96] showed good antibacterial activity against *Xoo* and *Xoc* with EC_50_ values of 38.74 μg/mL and 46.97 μg/mL, respectively. The preliminary mechanism of action of these compounds were explored, and it was found that these compounds could inhibit the production of exopolysaccharides of *Xoo* and increase the permeability of the cell membrane.

## 4. Insecticides Containing Phenoxypyridine Scaffold

### 4.1. Transient Receptor Potential Vanilloid Channel Blockers

**Figure 11:**
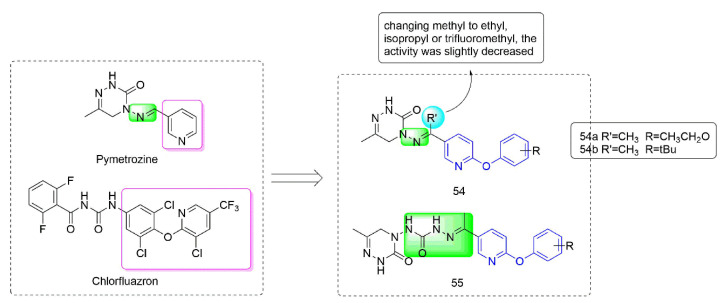
Transient receptor potential vanilloid channel blockers containing phenoxypyridine.

Pymetrozine [97] is a triazinone insecticide [98] that acts on the specific insecticide target protein transient receptor potential vanilloid (TRPV) ion channel, and showed no cross-resistance with other insecticides [99]. Compounds **54**–**55** (Figure 11) were developed by Nankai University with both phenoxypyridine groups and triazinone groups. The activity against aphids of Compound **54**, which was synthesized by Yang et al. [100] by constructing phenoxypyridine structure and introducing a methyl group to the imino group, was significantly improved. At the concentration of 5 mg/kg, the activities against aphids of **54a** (80%) and **54b** (80%) were both higher than those of pymetrozine (30%). Meanwhile, **54** also exhibited significant insecticidal activity against mosquitoes and lepidopteran pests (cotton bollworm, corn borer, and oriental stick insect). By modifying the linker arm, Wang et al. [101,102] designed and synthesized a series of triazinone derivatives **55** containing an acylhydrazone structure. These compounds had certain activities against aphids, cotton bollworm, corn borer, and armyworm.

### 4.2. Complex I Inhibitors

**Figure 12:**
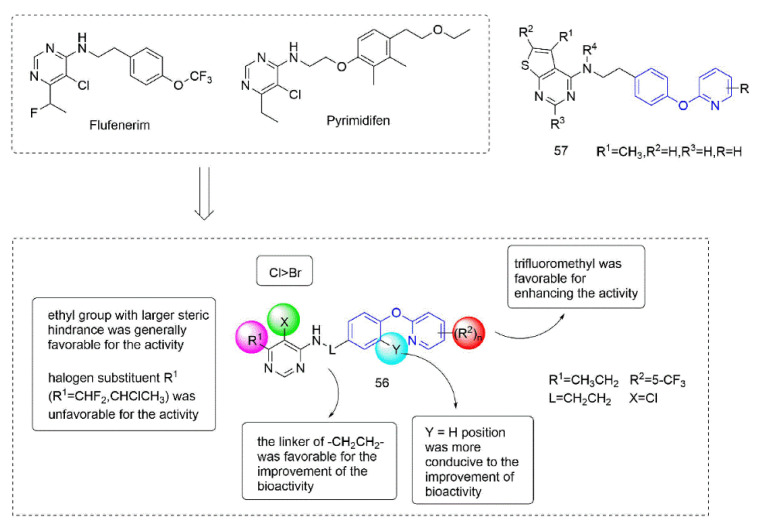
Complex I inhibitors (4-aminopyrimidine) containing phenoxypyridine.

Some insecticides and acaricides (flufenerim, purimidifen, tebufenpyrad, and tolfenpyrad [103]) worked by inhibiting the mitochondrial electron transport (MET) at complex I to disrupt respiration, known as complex I inhibitors [104]. Most of the 4-aminopyrimidine [105] derivatives that were synthesized by Wang et al. [106] through intermediate derivatization methods showed good activity against *Myzus persicae*, among which **56** (Figure 12) had the highest activity and the lowest LC_50_ value of 0.34 mg/L. The structure-activity relationships suggested that the linker of -CH_2_CH_2_- was favorable for bioactivity; the halogen substituent at the X position (X = Cl, Br) was more beneficial to the activity; for R^1^, the ethyl group with large steric resistance was generally conducive to improve the activity. The substituted thienopyrimidine amines **57** (Figure 12) that were synthesized by Chai et al. [107] had broad-spectrum insecticidal and acaricidal activity, which were very effective against lepidoptera pests, homoptera, and mites even at a very low dose, especially against aphids, *Tetranychus cinarcini*, *Plutella xylodes,* and armyworm.

**Figure 13:**
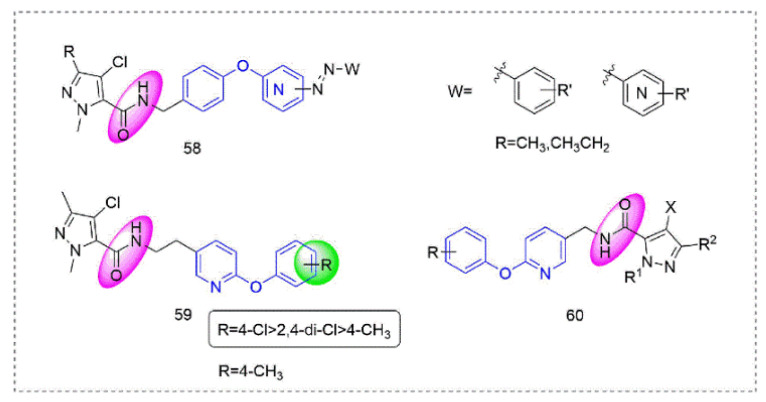
Complex I inhibitors (Pyrazole-5-carboxamide) containing phenoxypyridine.

Pyrazole-5-carboxamide insecticides **58** (Figure 13) containing an azo structure were synthesized by Shao et al. [108], many of which had 100% activity against *Aphis craccivora Koch* and *Tetranychus cinnabarinus*. Compound **59** [109] showed broad-spectrum insecticidal activity and a 100% mortality rate against *Plutella xylostella* and *Myzus persicae* at 600 mg/L. At the same time, several compounds had good activity against *Blumeria graminis* and southern corn rust. Pyrazole derivatives **60** that were designed and synthesized by Okada et al. [110] had good insecticidal activity against various insect pests (*Plutella xylostella*, *Nilaparvata lugens*, and the eggs and adults of *Tetranychus urticae*).

### 4.3. Other Insecticides

**Figure 14:**
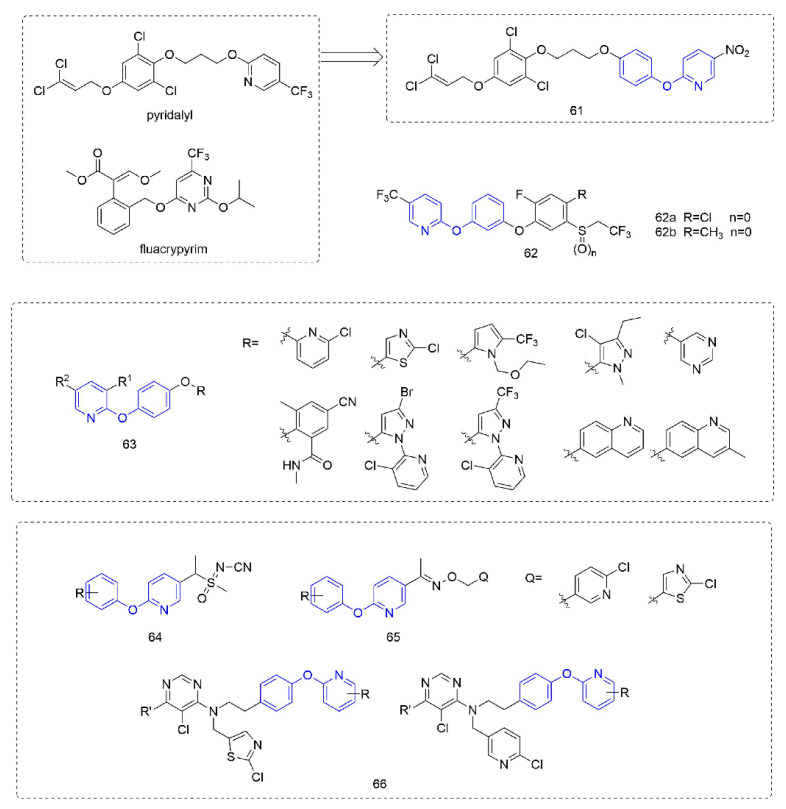
Other compounds with insecticidal activity containing phenoxypyridine.

Phenoxypyridine-containing compounds with insecticidal activity are summarized in Figure 14. Pyridalyl [111] inhibited cellular protein synthesis in insect cell lines but not mammalian cell lines. The novel dihalopropene ether insecticides that were synthesized by Liu et al. [112] exhibited good insecticidal activity. The LC_50_ of Compound **61**, which introduced phenoxypyridine, was 4.05 mg/L and 9.82 mg/L against *M. separate* and *P. litura*, respectively, was better than the control pyridalyl (LC_50_ = 4.81 mg/L and 10.07 mg/L) and better than the compounds with other aromatic ring substitutions. Alkylphenyl sulfide derivatives **62** that was reported by Kumiai Chemical Industry Co., Ltd. [113] had more than 90% control of *Tetranychus urticae (Koch)* at a concentration of 4 mg/L. Inspired by juvenile hormone, the analogues **63** that were prepared by Li et al. [114] with the introduction of phenoxypyridine were more than 85% effective against *Nilaparvata lugens* at a concentration of 200 mg/L. Using phenoxypyridine molecular plug-ins, the sulfoximine and oxime ether, Compounds **64** and **65** with insecticidal activity were synthesized by Liu et al. [115] and Du et al. [116]. The neonicotinoids **66** that was designed and synthesized by Tang et al. [117] had certain activities against lepidoptera, homoptera, coleoptera, and the larvae and adults of orthoptera.

## 5. Conclusions

In pesticide applications, phenoxypyridine played an important role in the development of lead compounds. Compounds that were derived by linking phenoxypyridine to different active fragments or changing the substituents of phenoxypyridine exhibited a wide range of biological activity, such as herbicidal, fungicidal, bactericidal, and insecticidal activities. In this paper, the derivatives with different activities were classified. The summary of the structure-activity relationship of the derivatives indicated that structural modifications at different positions of phenoxypyridine could improve its activity. Previous studies had focused on compounds that were linked to the phenoxy group at position 2 of pyridine, possibly due to the difficulty of synthesis, so the relationship between the position of the N atom on pyridine and biological activity was unclear. The inhibitory effects of these compounds may be performed by different mechanisms and, therefore, further studies on the mechanism (or targets) are necessary for better evaluations. Still, a lot of activity of phenoxypyridine needs to be prospected in bactericides. In conclusion, phenoxypyridine could be considered as the promising active scaffold for pesticides.

## Figures and Tables

**Table 1:** Comparison of the activity of diphenyl ethers and phenoxypyridine pesticides.

No.	Pesticides Containing Diphenyl Ether		Compounds Containing Phenoxypyridine	
	Structure	Activity	Structure	Activity
1	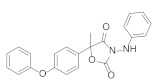 Famoxadone [9]	*Rhizoctonia solani* IC_50_ > 100 mg/L *Pythium aphanidermatum* IC_50_ > 100 mg/L *Pyricularia grisea* IC_50_ > 100 mg/L *Phomopsis asparagi* IC_50_ > 100 mg/L	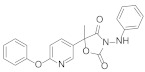 A [9]	*Rhizoctonia solani* IC_50_ = 6.53 mg/L *Pythium aphanidermatum* IC_50_ = 8.62 mg/L *Pyricularia grisea* IC_50_ = 11.46 mg/L *Phomopsis asparagi* IC_50_ = 16.2 mg/L
2	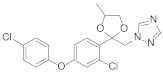 Difenoconazole [10]	*Rhizoctonia solani* EC_50_ = 8.93 mg/L *Pyricularia oryae* EC_50_ = 2.42 mg/L *Gibberella zeae* EC_50_ = 4.40 mg/L *Botrytis cinerea* EC_50_ > 20 mg/L	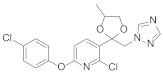 B [11]	*Botrytis cinerea* ED_50_ = 2.7 mg/L *Septoria tritici* ED_50_ = 0.008 mg/L *Pyricularia oryzae* ED_50_ = 1.2 mg/L
			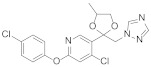 C [12]	*Botrytis cinerea* ED_50_ = 8.9 mg/L *Septoria tritici* ED_50_ = 0.013 mg/L *Pyricularia oryzae* ED_50_ = 9.5 mg/L
3	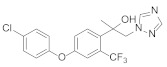 Mefentrifluconazole [13]	30% in vivo protective activity against *Sphaerootheca* at 10 mg/L	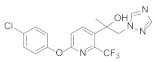 D [14]	100% in vivo protective activity against *Sphaerootheca* at 10 mg/L
4	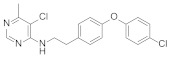 E [15]	EC_50_ = 8.62 mg/L	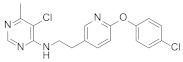 F [15]	EC_50_ = 0.19 mg/L
5	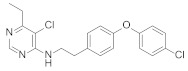 G [15]	Cucumber Downy Mildew EC_50_ = 6.25–25 mg/L	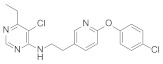 H [15]	Cucumber Downy Mildew EC_50_ = 2.65 mg/L

## Data Availability

Not applicable.
